# Supplementary material for: Phosphatidic Acid Reverses Obesity Induced by a High-Fat, High-Sugar Diet at the Transcriptional Level
Source: Genes (Basel). 2025 Sep 19;16(9):1112. doi: 10.3390/genes16091112 (PMC12469495; doi:10.3390/genes16091112)
Supplement: Supplementary file 1 [file genes-16-01112-s001.zip › Supplemantary Material - revised.pdf]

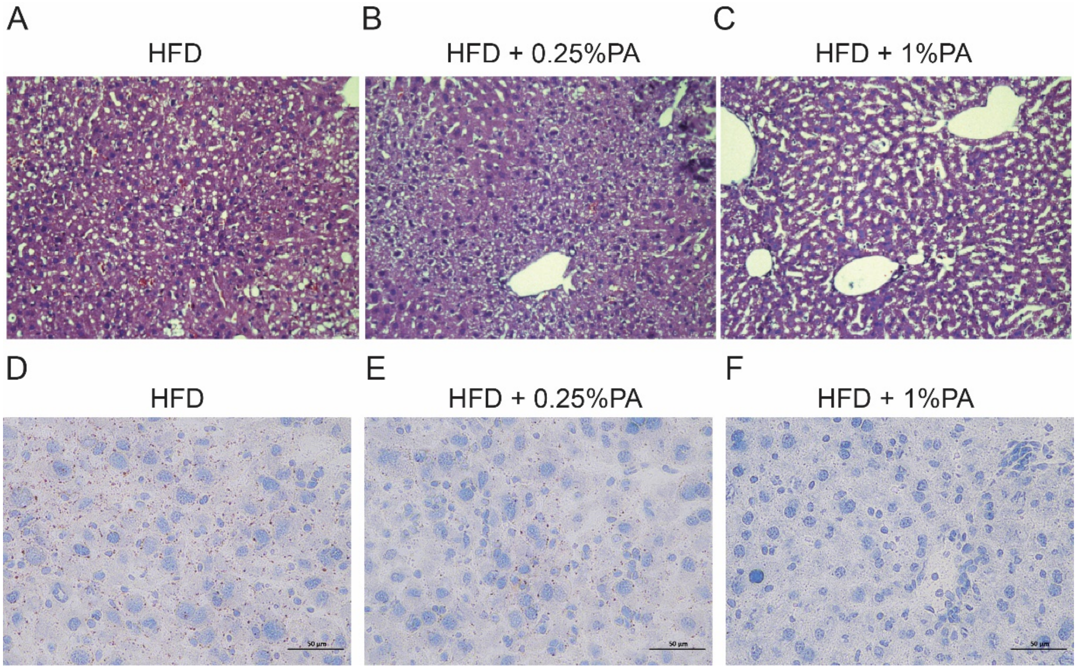

FigureS1

PA did not affect liver immunohistochemistry but reduced the storage of lipid droplets.

Liver paraffin sections stained with H&E from mice fed with HFD (A), HFD + 0.25% PA (B) and HFD + 1% PA (C) all exhibited vacuolar structures, indicating hepatic steatosis. Oil Red O staining of liver sections revealed the lipid droplet storage status in mice from the HFD (D), HFD + 0.25% PA (E) and HFD + 1% PA (F) groups.

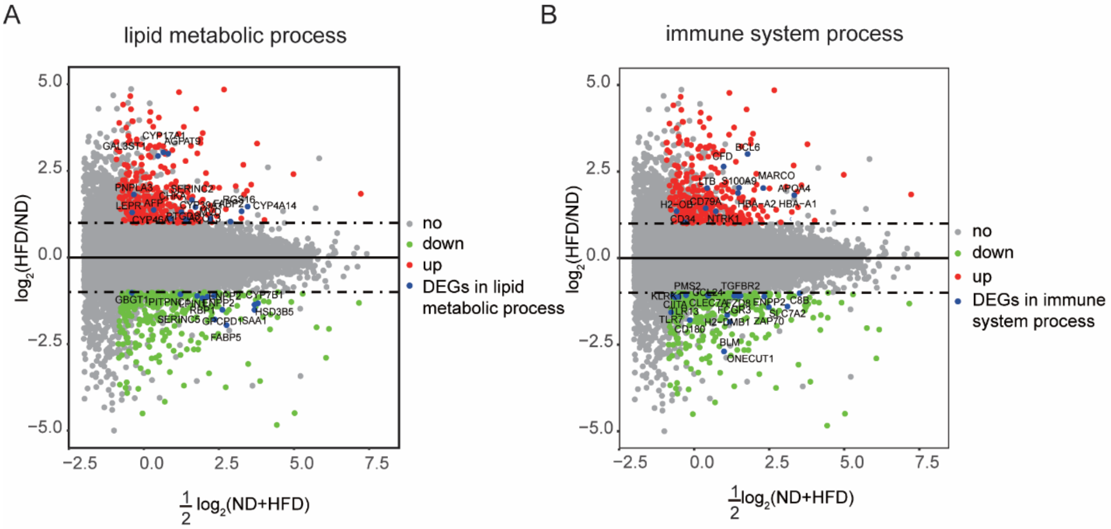

Figure S2

HFD affects gene expression changes in lipid metabolism and immune pathways at the transcriptional level.

In the MA plots of differentially expressed genes between the ND and HFD groups, genes enriched in the lipid metabolism process (A) and genes enriched in the immune system process (B) are highlighted.

Supplementary Table1

FPKM of Genes have significantly reversed expression levels by PA.
